# Thromboxane A2-Driven Vascular Hyperreactivity in Cadmium-Induced Hypertension: Role of Oxidative Stress and Therapeutic Implications

**DOI:** 10.3390/jcm15166273

**Published:** 2026-08-13

**Authors:** Miguel A. García-González, Gustavo López-López, Fausto Atonal-Flores, Celeste Santamaría, Eduardo Brambila, Samuel Treviño, Alfonso Díaz, Victor E. Sarmiento-Ortega, Jorge Flores, Jose L. Flores-Guerrero

**Affiliations:** 1 Institute of Physiology, Autonomous University of Puebla, Puebla 72592, Mexico; 2 Institute of Neurobiology, National Autonomous University of Mexico, Juriquilla 76230, Mexico; 3 Faculty of Chemistry Sciences, Autonomous University of Puebla, Puebla 72592, Mexico; 4 Faculty of Medicine, Autonomous University of Puebla, Puebla 72420, Mexico; 5 Interdisciplinary Center for Research and Science Education, Autonomous University of Puebla, Puebla 72570, Mexico; 6 MRC Unit for Lifelong Health and Ageing, University College London, London WC1E 7HB, UK

**Keywords:** hypertension, cadmium, thromboxane, aorta, planetary health

## Abstract

**Background/Objectives**: Environmental factors, including heavy metals such as cadmium, are increasingly recognized as important contributors to hypertension beyond traditional risk factors. To date, the role of angiotensin and alpha-adrenergic receptors in hypertension induced by cadmium exposure has been explored. However, the involvement of thromboxane A2 receptors in vascular hyperreactivity and hypertension in rats with chronic cadmium administration is unknown. **Methods**: This study aimed to evaluate changes in vascular reactivity due to the action of thromboxane A2 in isolated aortas from hypertension induced by chronic administration of cadmium (HICAD) rats and whether this effect is associated with changes in the redox balance. Aortas were homogenized in phosphate buffer to assess oxidative stress and vascular reactivity. MDA, 4-HDAs, GPx, and GR were quantified spectrophotometrically. Aortic rings were used for vasodilator and vasoconstrictor responses, evaluating NADPH oxidase involvement using apocynin. **Results**: We found that in aortas from HICAD rats there was: (1) a significant increase in malondialdehyde and 4-hydroxyalkenals (2 to 4-fold), as well as a significant reduction in glutathione reductase (~50%), and (2) a significant 35% increase in the vasoconstrictor response to U46619 (thromboxane A2 analogue), but no significant changes (~2%) when incubated with an NADPH oxidase inhibitor. **Conclusions**: The increase in the vasoconstrictor response to thromboxane A2 can be measured by the increase in oxidative stress and the involvement of NADPH oxidase.

## 1. Introduction

Over the past years, hypertension has remained a major risk factor for cardiovascular mortality [1]. Recently, environmental factors such as heavy metals have been recognized as important stressors that may contribute to the development of hypertension beyond traditional risk factors [2]. Cadmium (Cd^2+^) is a heavy metal and environmental pollutant that is associated with significant public health concerns. Its harmful effects are primarily attributed to increased production and excessive release of reactive oxygen species (ROS), enhanced lipid peroxidation, and impaired antioxidant defence mechanisms [3,4,5].

Vascular hyperreactivity is a hallmark of hypertension, with thromboxane A_2_ emerging as a key mediator linking oxidative stress to enhanced vasoconstrictor responses. Exposure to Cd^2+^ is particularly concerning due to its efficient absorption, widespread systemic distribution, and the frequent exceedance of recommended safety limits. The toxic effects of Cd^2+^ are largely mediated by increased ROS production, enhanced lipid peroxidation, and disruption of endogenous antioxidant defences [6,7,8,9,10].

In this context, oxidative stress has been proposed as a central mechanism underlying endothelial dysfunction and elevated blood pressure following chronic Cd^2+^ exposure [11]. Experimental and clinical evidence suggests that excessive ROS disrupt vascular homeostasis by reducing nitric oxide bioavailability and promoting vasoconstriction. Among the enzymatic sources of ROS, NADPH oxidase has been identified as a major contributor within the vascular wall and plays a critical role in the development of endothelial dysfunction in several cardiovascular diseases [12,13,14,15].

Previous studies using isolated aortic rings from hypertensive rats chronically exposed to Cd^2+^ have demonstrated increased oxidative stress and activation of NADPH oxidase. However, despite the established involvement of vasoconstrictor pathways in hypertension, the specific contribution of thromboxane A_2_ to vascular hyperreactivity in this model remains unclear. Understanding this interaction may provide new insights into the mechanisms linking environmental toxicants, oxidative stress, and vascular dysfunction, while also identifying potential therapeutic targets for hypertension management [16,17,18].

Previous reports have shown that contractile agonists induce vascular hypercontraction; however, incubation of aortic rings with apocynin, a specific NADPH oxidase inhibitor, exerts a protective effect against this hypercontractile response [18]. Nevertheless, the involvement of thromboxane A_2_ (TXA_2_) receptors in the regulation of vascular tone in HICAD aortas has not yet been investigated. TXA_2_ and its G protein-coupled receptor have been implicated in the pathogenesis of cardiovascular disorders, including systemic hypertension [19,20,21]. Therefore, investigating the role of TXA_2_ is essential for understanding the mechanisms through which Cd^2+^ alters vascular function. Accordingly, the aim of this study was to determine the involvement of TXA_2_ in the hypercontractility of aortas following sustained cadmium exposure and to evaluate whether this effect is mediated by oxidative imbalance.

## 2. Materials and Methods

### 2.1. Experimental Models

Male Wistar rats weighing approximately 200 g were obtained from the same institution. Animals were maintained under controlled temperature and humidity conditions, with a 12-h light/dark cycle and ad libitum access to food and water. The experimental protocol approved by the Bioethics Committee of the Benemérita Universidad Autónoma de Puebla (ID: 00220) was conducted in accordance with internationally recognized guidelines for the ethical use and care of laboratory animals. All procedures complied with the principles established in the Guide for the Care and Use of Laboratory Animals published by the National Research Council, as well as the ARRIVE (Animal Research: Reporting of In Vivo Experiments) guidelines and the principles of Replacement, Reduction, and Refinement (3Rs) for animal experimentation. Furthermore, the study adhered to the Official Mexican Standard NOM-062-ZOO-1999, ensuring appropriate housing conditions, minimization of animal suffering, adequate anesthesia and euthanasia procedures, and the implementation of methodological strategies aimed at maintaining scientific rigor and animal welfare throughout the experimental protocol.

Cadmium chloride (CdCl_2_; J.T. Baker, Phillipsburg, NJ, USA) was administered through drinking water at concentrations of 40 or 80 ppm for 8 weeks. Rats were randomly assigned, using the digital randomizer available at graphpad.com, into three experimental groups: control (CTRL), CdCl_2_ 40 ppm (Cd^2+^/40 ppm), and CdCl_2_ 80 ppm (Cd^2+^/80 ppm).

Group allocation was generated by an investigator not involved in data collection or analysis. During allocation, the investigator assigning animals to the experimental groups was aware of group identities, whereas personnel responsible for conducting the experiments were blinded to treatment allocation whenever feasible. Outcome assessments were performed by investigators blinded to group assignment. Data analysis was conducted using coded datasets, and the analyst remained blinded to group identities until completion of all analyses.

The selection of the number of animals was based on previous experimental studies evaluating vascular reactivity, oxidative stress, and blood pressure alterations in models of chronic Cd^2+^ exposure and hypertension, and therefore an a priori power calculation was not performed [16,17,18]. The number of animals included in each group was considered sufficient to detect biologically relevant differences while minimizing unnecessary animal use, in accordance with the principles of Reduction and Refinement established by international guidelines for animal experimentation. Group sizes were selected to provide adequate experimental reproducibility and statistical reliability for vascular and biochemical analyses, while also accounting for the expected biological variability inherent to in vivo cardiovascular studies. Furthermore, the selected sample size was consistent with recommendations of the ARRIVE guidelines and the Guide for the Care and Use of Laboratory Animals, ensuring an appropriate balance between scientific rigor, ethical considerations, and animal welfare.

### 2.2. Blood Pressure

Systemic blood pressure was measured at the beginning and end of the experimental period using a non-invasive tail-cuff plethysmography system (NIBP Controller, ADInstruments; Colorado Springs, CO, USA), as previously described [22,23]. This method estimates systolic blood pressure by detecting changes in tail blood flow during gradual cuff inflation and deflation in conscious animals. Prior to the experimental measurements, rats underwent a 7-day acclimatization and conditioning period to minimize stress-induced variability. During this period, animals were placed daily in restraining chambers for short intervals under controlled temperature and lighting conditions in order to habituate them to handling and restraint procedures.

On the day of evaluation, animals were maintained under the same environmental conditions in a quiet, temperature-controlled room to promote peripheral vasodilation and ensure adequate tail blood flow [24]. Rats were allowed to stabilize before measurements were initiated. The occlusion cuff and pulse sensor were placed around the tail according to the manufacturer’s instructions, and multiple consecutive measurements were obtained for each animal to improve reliability and reduce variability. Measurements presenting excessive movement artifacts or unstable pulse detection were excluded from the analysis. The average of the valid recordings was used as the final systolic blood pressure value for each animal. Data acquisition, visualization, and storage were performed using LabChart version 7.0 software (ADInstruments; Colorado Springs, CO, USA).

For blood collection by cardiac puncture and thoracic aorta extraction, animals were anesthetized with a ketamine-xylazine mixture (60 and 4 mg/kg body weight, respectively), ensuring the absence of pain and distress during euthanasia. Adequate anesthesia was confirmed by the absence of retro-orbital sinus and paw withdrawal reflexes. Animals were subsequently euthanized by respiratory arrest during thoracotomy.

### 2.3. Biochemical Assays

For biochemical analyses, aortic tissues from each rat were mechanically homogenized in potassium phosphate buffer (0.05 M, pH 7.8). All reagents used for the quantification of biochemical markers were purchased from Sigma-Aldrich (St. Louis, MO, USA).

### 2.4. Lipoperoxidation Markers

For total protein quantification using the Lowry method, homogenates were centrifuged at 10,000× *g* for 15 min at 4 °C. The concentrations of malondialdehyde (MDA) and 4-hydroxyalkenals (4-HDAs) were subsequently determined in the supernatant by spectrophotometric analysis at 586 nm.

### 2.5. Enzymatic Activity

Glutathione peroxidase (GPx) activity was determined using a freshly prepared phosphate buffer containing 50 mM K_2_HPO_4_ (pH 7.1), 1 mM EDTA, 1 mM catalase inhibitor, 0.2 mM NADPH, 1 unit of glutathione reductase (GSR), and 1 mM reduced glutathione (GSH). Subsequently, 0.1 mL of the homogenate fraction was added to the working buffer and incubated for 5 min at room temperature. Hydrogen peroxide (0.25 mM; 0.1 mL) was then added, and the mixture was centrifuged at 13,000 rpm for 15 min at 4 °C. GPx activity was quantified in the resulting supernatant by spectrophotometric analysis at 340 nm.

Glutathione reductase (GR) activity was measured using potassium phosphate buffer (139 mM, pH 7.4). The buffer was mixed with the tissue homogenate and centrifuged at 12,000 rpm for 15 min at 4 °C. The recovered supernatant was then combined with NADPH (2.5 mmol/mL) and FAD (315 µmol/L). After 3.3 min, the initial absorbance was measured at 340 nm. Glutathione disulfide (GSSG, 22 mmol/L) was subsequently added, and absorbance was measured again after 2.9 min at the same wavelength. Enzymatic activity was calculated from the difference between absorbance values.

### 2.6. Aortic Vascular Reactivity

The thoracic aorta was carefully dissected and cleaned of surrounding fat and connective tissue, and then sectioned into 3-mm rings. The rings were mounted between two stainless-steel wires in an antiparallel configuration within 5 mL organ baths containing Krebs–Henseleit solution composed of NaCl (118 mM), KCl (4.75 mM), NaHCO_3_ (25 mM), CaCl_2_ (1.2 mM), KH_2_PO_4_ (1.2 mM), and glucose (11 mM). Preparations were maintained at 37 °C under continuous aeration with 95% O_2_ and 5% CO_2_. Isometric tension was applied using a micromanipulator, and contractile responses were recorded using a GRASS FT-03 transducer coupled to a GRASS 7-DAJK amplifier (Astro-Med, Inc., West Warwick, RI, USA) and analyzed with LabChart 7.0 software (ADInstruments).

Endothelium-dependent vasodilator function was evaluated by constructing concentration–response curves (CRCs) to acetylcholine (ACh) in aortic rings precontracted with phenylephrine (Phe, 1 × 10^−6^ M), as previously described [25,26]. Vasoconstrictor responses were assessed by CRCs generated with phenylephrine and the thromboxane A_2_ (TXA_2_) analog U46619. Contractile responses were normalized to the maximal contraction induced by KCl (80 mM). For all CRCs, cumulative agonist concentrations ranging from 1 × 10^−9^ to 3 × 10^−5^ M were used. To investigate the involvement of NADPH oxidase, selected aortic rings were incubated with apocynin (APO, 300 µmol/L) for 15 min prior to CRC construction. All drugs were obtained from Sigma-Aldrich (St. Louis, MO, USA).

### 2.7. Statistical Analyses

The pharmacological data for CRC, maximum effect (Emax), and logarithm of effective concentration 50 (pD2) were obtained using the logistic equation in version 8.5 of Origin Lab software. Baseline and week 8 measurements of body weight, blood glucose, and systolic blood pressure were obtained from the same animals; therefore, these variables were analyzed using paired statistical tests for within-group comparisons, whereas comparisons among experimental groups at each time point were performed using the appropriate between-group tests. For vascular reactivity experiments, the experimental unit was the individual animal. Concentration–response curves (CRCs) were generated from isolated aortic rings obtained from each animal, and responses at each agonist concentration were used exclusively to fit a sigmoidal concentration–response model by nonlinear regression. The estimated maximal response (Emax) and sensitivity (pD_2_) were then calculated for each individual animal, and these derived parameters were used for subsequent statistical comparisons among experimental groups. Accordingly, statistical comparisons were based on Emax and pD_2_ values rather than on repeated observations across the entire concentration–response curves, thereby avoiding pseudoreplication arising from multiple measurements obtained from the same preparation. All results were expressed as mean ± SEM. One-way analysis of variance (ANOVA) coupled with a Bonferroni test was used to compare multiple groups, and values of *p* < 0.05 were considered statistically significant. Distribution of residuals were evaluated to confirm the assumptions of ANOVA testing.

## 3. Results

### 3.1. Effects of Chronic Cadmium Exposure on Body Weight, Blood Glucose, and Blood Pressure

Animals exposed to chronic Cd^2+^ administration for 8 weeks exhibited marked metabolic and hemodynamic alterations compared with the control group. Although body weight increased over time in all experimental groups, Cd^2+^-treated animals showed significantly reduced weight gain. The weight of control animals increased from 235.3 ± 3.7 g to 419.5 ± 3.1 g, whereas animals exposed to Cd^2+^ reached final body weights of only 380.9 ± 5.8 g (40 ppm) and 379.3 ± 9.6 g (80 ppm), representing an approximate reduction of 40 g compared with controls.

In parallel, Cd^2+^ exposure induced a significant dose-dependent increase in blood glucose levels. While control animals maintained relatively stable glucose concentrations (95.7 ± 1.2 to 98.5 ± 0.9 mg/dL), Cd^2+^-treated groups exhibited increases to 110.0 ± 1.0 mg/dL (40 ppm) and 115.5 ± 0.8 mg/dL (80 ppm), corresponding to elevations of approximately 15–20 mg/dL.

Moreover, chronic Cd^2+^ administration produced a pronounced increase in systolic blood pressure, confirming the development of a hypertensive phenotype. In contrast to control animals, which showed no significant changes in systolic pressure (102.9 ± 1.8 to 102.4 ± 1.7 mmHg), Cd^2+^-treated rats exhibited dose-dependent elevations to 121.7 ± 3.2 mmHg (40 ppm) and 133.1 ± 1.4 mmHg (80 ppm), representing increases greater than 20–30 mmHg after 8 weeks of exposure (Table 1).

### 3.2. Reduction in Acetylcholine-Dependent Relaxation in HICAD Aortas

**Figure 1:**
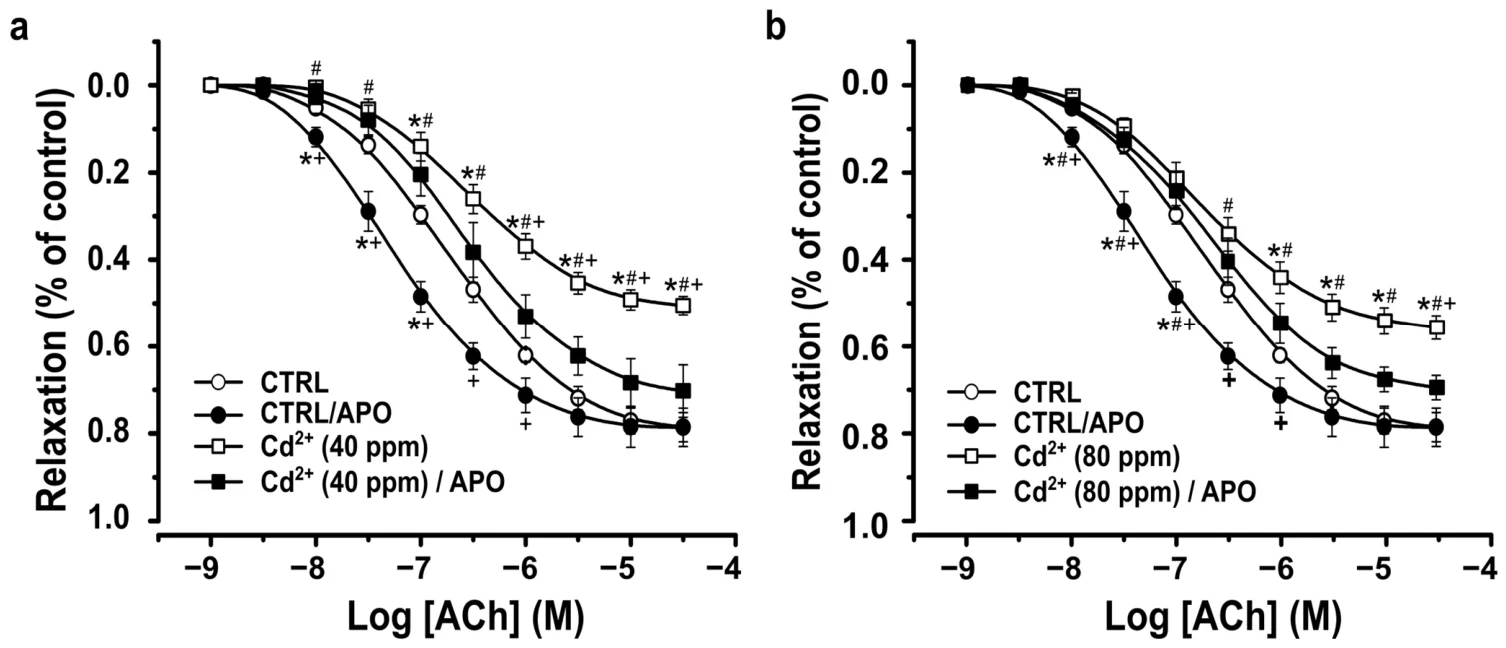
Cadmium induces decreased relaxant capacity in rat aortas. (**a**) Effect of acetylcholine (ACh) on relaxant capacity in aortic rings chronically exposed to 40 ppm cadmium. (**b**) Effect of chronic cadmium exposure (80 ppm) on relaxant capacity assessed with cumulative ACh concentrations in aortic rings. Results are presented as mean ± SEM (n = 6). * indicates *p* < 0.05 vs. CTRL; # indicates *p* < 0.05 vs. CTRL/APO; + indicates *p* < 0.05 vs. Cd^2+^/APO. The *p* value was calculated using one-way ANOVA coupled to the Bonferroni test. (M) indicates log molar concentration.

It was observed that the concentration–response curves (CRCs) to acetylcholine (ACh) in aortic rings from hypertensive animals treated with Cd^2+^ (40 or 80 ppm) exhibited a reduced maximal response (Emax) compared with aortic rings from the CTRL group, whereas no significant changes were detected in pD_2_ values (Figure 1 and Table 2). In contrast, incubation of aortic rings from hypertensive animals with apocynin (APO) abolished these alterations, as no significant differences in either Emax or pD_2_ were observed compared with the CTRL group (Figure 1 and Table 2).

### 3.3. Hypercontraction Induced by Thromboxane A2 and Phenylephrine in the Aortas of HICAD

**Figure 2:**
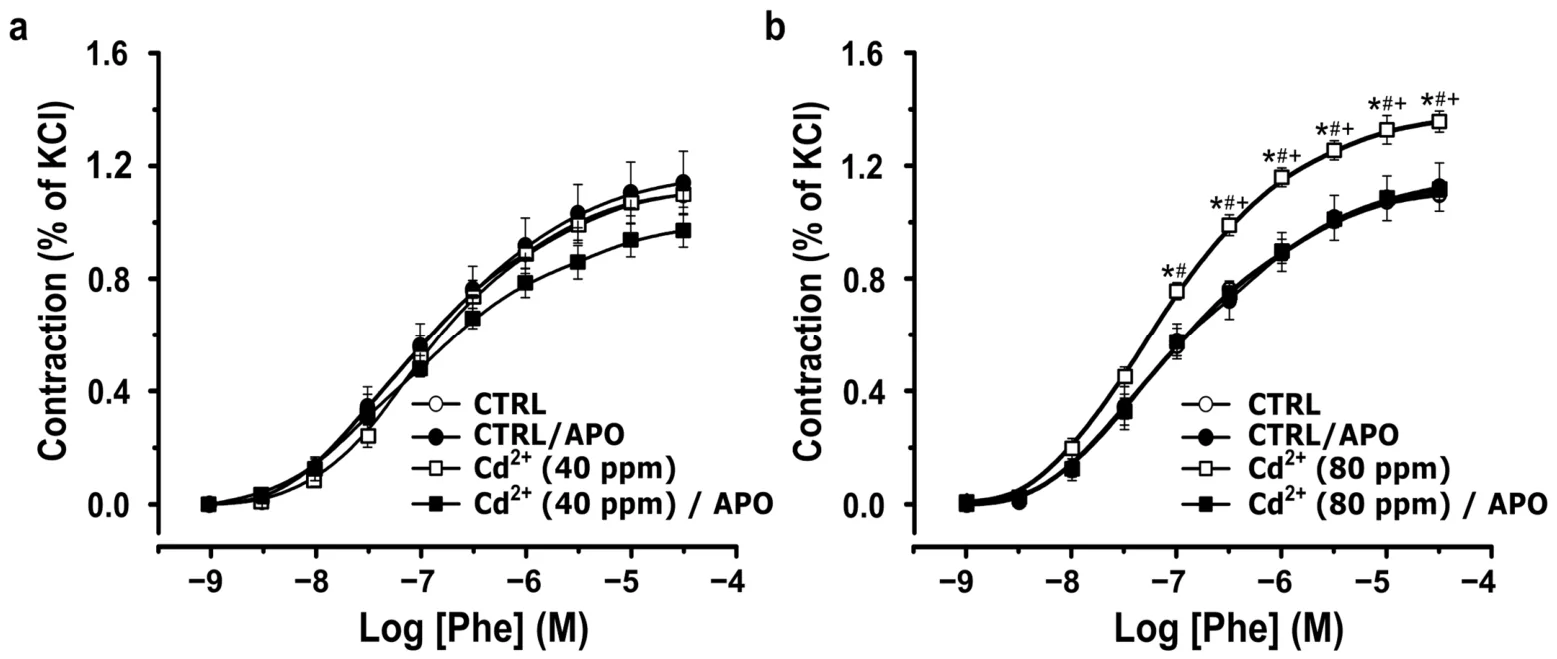
High cadmium concentration increases the contractile response to phenylephrine. (**a**) Effect of phenylephrine (Phe) on contraction in aortic rings chronically exposed to cadmium (40 ppm). (**b**) Effect of chronic administration of 80 ppm cadmium on contraction in aortic rings exposed to cumulative concentrations of Phe. Results are expressed as mean ± SEM (n = 6). * indicates *p* < 0.05 vs. CTRL; # indicates *p* < 0.05 vs. CTRL/APO; + indicates *p* < 0.05 vs. Cd^2+^/APO. The *p* value was calculated using one-way ANOVA coupled to the Bonferroni test. (M) indicates log molar concentration.

**Figure 3:**
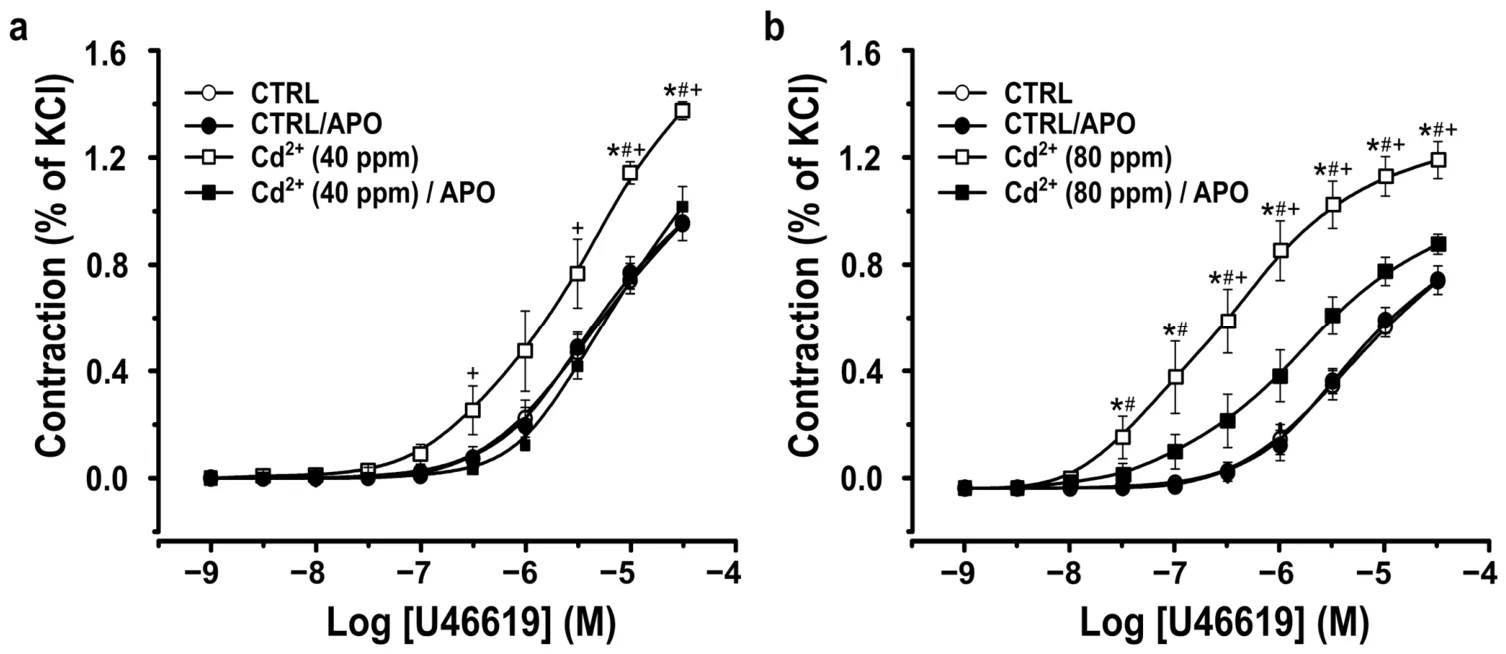
Thromboxane (U46619) elicits contractile hyperresponsiveness of aorta exposed to cadmium. Chronic cadmium administration induces increased contractile capacity to thromboxane of aortic rings at 40 (**a**) and 80 ppm (**b**). Results are expressed as mean ± SEM (n = 6). * indicates *p* < 0.05 vs. CTRL; # indicates *p* < 0.05 vs. CTRL/APO; + indicates *p* < 0.05 vs. Cd^2+^/APO. The *p* value was calculated using one-way ANOVA coupled to the Bonferroni test. (M) indicates log molar concentration.

The concentration–response curves (CRCs) to phenylephrine (Phe) and TXA_2_ in aortic rings obtained from hypertensive animals demonstrated a dose-dependent increase in contractile responses according to the concentration of Cd^2+^ exposure (Figure 2 and Figure 3).

The Phe CRCs obtained from HICAD animals exposed to 40 ppm Cd^2+^ showed no significant differences in either Emax or pD_2_ compared with aortic rings from the CTRL group (Table 2). In contrast, aortic rings from HICAD animals exposed to 80 ppm Cd^2+^ exhibited a significant increase in Emax without changes in pD_2_ relative to CTRL animals. Incubation with apocynin (APO) abolished these alterations, as Phe CRCs from aortic rings of HICAD animals treated with either 40 or 80 ppm Cd^2+^ showed no significant differences in Emax or pD_2_ compared with the CTRL group (Figure 2).

Similarly, CRCs for the TXA_2_ analog U46619 in aortic rings from hypertensive animals exposed to 40 or 80 ppm Cd^2+^ showed a significant increase in Emax, whereas pD_2_ values remained unchanged compared with CTRL animals (Table 2). Notably, aortic rings from animals exposed to 80 ppm Cd^2+^ displayed a greater Emax than those exposed to 40 ppm Cd^2+^, indicating a concentration-dependent enhancement of TXA_2_-mediated vasoconstriction.

Furthermore, incubation with APO normalized the contractile response in aortic rings from hypertensive animals exposed to 40 ppm Cd^2+^, with no significant differences in Emax or pD_2_ compared with CTRL preparations. In aortic rings from animals exposed to 80 ppm Cd^2+^, APO significantly reduced Emax without affecting pD_2_ when compared with non-incubated HICAD (80 ppm) rings (Figure 3).

### 3.4. Alterations in Lipoperoxidation Concentrations and Enzymatic Antioxidant Systems in the Aortas of HICAD Rats

**Figure 4:**
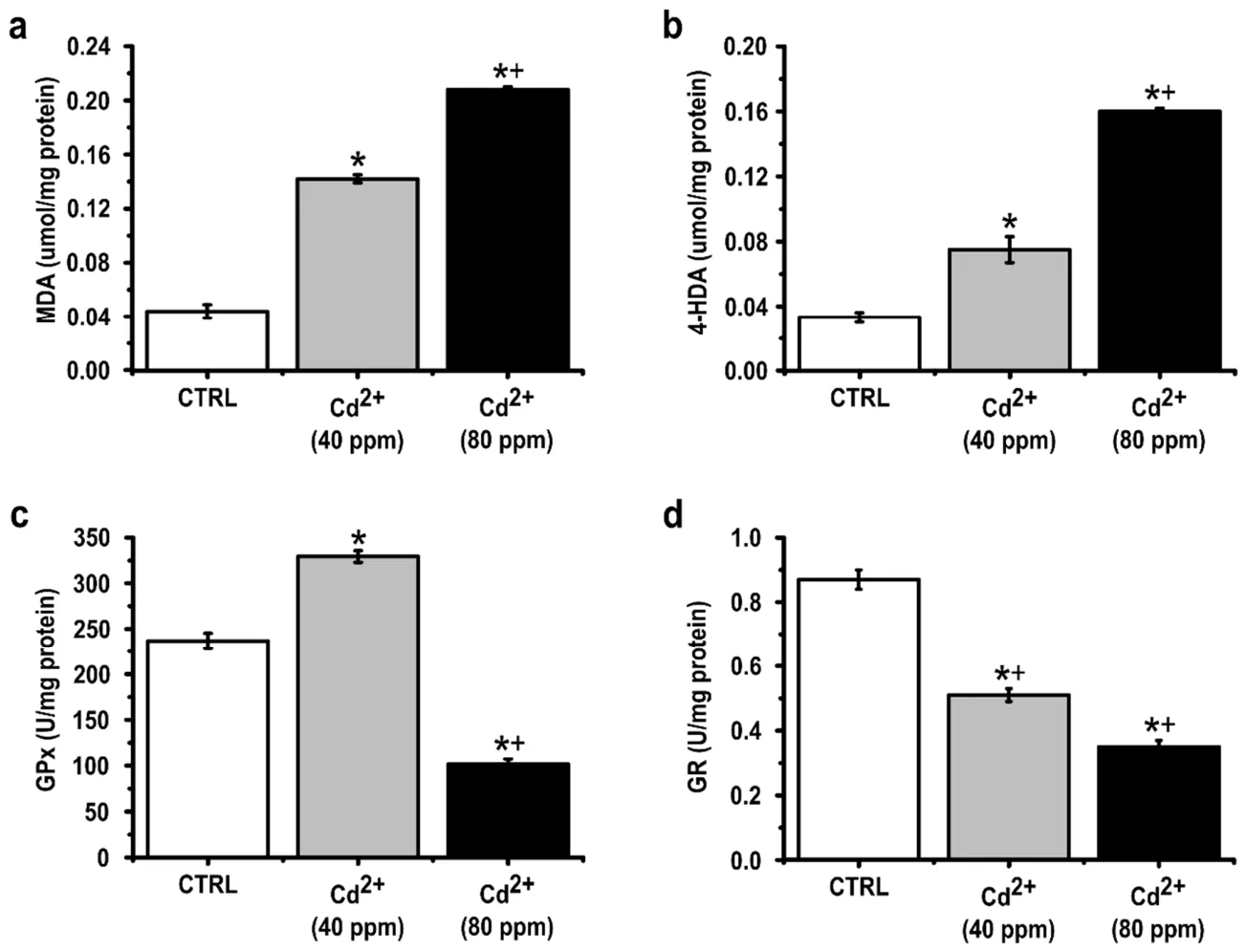
Cadmium increases lipoperoxidation and modifies enzymatic antioxidants. (**a**) Malondialdehyde (MDA) level increases in a cadmium concentration-dependent manner. (**b**) 4-hydroxyalkenal (4-HDA) level increases according to the administered dose of cadmium. (**c**) Glutathione peroxidase (GPx) activity increases at low doses but decreases at high doses of cadmium. (**d**) Glutathione reductase (GR) activity decreases in a cadmium concentration-dependent manner. Results are expressed as mean ± SEM (n = 8). * indicates *p* < 0.05 vs. CTRL; + indicates *p* < 0.05 vs. Cd^2+^ (40 ppm). The *p* value was calculated using one-way ANOVA coupled to the Bonferroni test.

Aortic tissue from HICAD animals exhibited a marked redox imbalance (Figure 4). Treatment with 40 ppm Cd^2+^ significantly increased malondialdehyde (MDA) concentrations compared with the CTRL group (0.04 ± 0.005 vs. 0.142 ± 0.003 µmol/mg, respectively). Moreover, animals exposed to 80 ppm Cd^2+^ showed even higher MDA levels than those treated with 40 ppm Cd^2+^ (0.208 ± 0.002 vs. 0.142 ± 0.003 µmol/mg, respectively), indicating a dose-dependent increase in lipid peroxidation.

Similarly, 4-hydroxyalkenal (4-HDA) concentrations were significantly elevated in the 40 ppm Cd^2+^ group compared with CTRL animals (0.075 ± 0.008 vs. 0.033 ± 0.003 µmol/mg, respectively). Exposure to 80 ppm Cd^2+^ further increased 4-HDA levels compared with the 40 ppm group (0.160 ± 0.002 vs. 0.075 ± 0.008 µmol/mg, respectively).

In contrast, glutathione reductase (GR) activity was significantly reduced in Cd^2+^-treated animals. Aortas from the 40 ppm Cd^2+^ group exhibited lower GR activity than the CTRL group (0.51 ± 0.02 vs. 0.87 ± 0.03 U/mg, respectively), whereas the 80 ppm Cd^2+^ group showed an additional reduction compared with the 40 ppm group (0.35 ± 0.02 vs. 0.51 ± 0.02 U/mg, respectively).

Regarding glutathione peroxidase (GPx), aortas from hypertensive rats exposed to 40 ppm Cd^2+^ displayed higher GPx activity compared with the CTRL group (329.3 ± 6.3 vs. 236.7 ± 8.5 U/mg, respectively). However, exposure to 80 ppm Cd^2+^ resulted in a marked reduction in GPx activity (101.8 ± 5.6 U/mg), which was significantly lower than that observed in both the CTRL and 40 ppm Cd^2+^ groups.

## 4. Discussion

Chronic exposure to Cd^2+^ has been reported to induce endothelial dysfunction and promote the development of hypertension [16,17,18]. However, the underlying mechanisms responsible for this endothelial dysfunction have not yet been fully elucidated. Previous studies have shown that angiotensin II and norepinephrine induce vascular hyperreactivity and hypertension during chronic Cd^2+^ exposure [17,18], effects associated with increased production of reactive oxygen species, mainly mediated by dysregulation of NADPH oxidase and COX-2 [27].

Among the mediators involved in vascular vasoconstriction, thromboxane A_2_ (TXA_2_) has been implicated in cardiovascular dysfunction; however, its contribution to vascular reactivity during chronic Cd^2+^ intoxication has not been previously described. The present study demonstrates for the first time that TXA_2_ induces hypercontractility in aortas from HICAD animals, an effect associated with oxidative stress, as evidenced by its reversal following NADPH oxidase inhibition.

The increase in blood glucose observed in Cd^2+^-treated groups is consistent with previous clinical [28,29,30] and experimental studies [31,32]. Hyperglycaemia has been attributed to pancreatic β-cell damage, impaired insulin secretion [32,33], reduced insulin sensitivity, and increased insulin resistance [34]. A limitation of the present study is the lack of experiments investigating the mechanisms responsible for the observed hyperglycaemia. Chronic Cd^2+^ exposure also produced a dose-dependent increase in blood pressure, consistent with previous reports linking Cd^2+^ toxicity to hypertension, endothelial dysfunction, and oxidative stress [27,35,36].

To investigate the mechanisms underlying Cd^2+^-induced hypertension, vascular reactivity was assessed in isolated aortic rings. HICAD animals exhibited impaired ACh-induced relaxation, supporting previous evidence that Cd^2+^ enhances ROS production through activation of NADPH oxidase and COX-2 [17,27,37]. Increased oxidative stress reduces nitric oxide (NO) bioavailability, impairs endothelium-dependent relaxation, and promotes vascular contraction [27,38]. Consistent with this mechanism, apocynin restored endothelial function, supporting previous observations that oxidative stress and reduced NO bioavailability are major contributors to Cd^2+^-induced endothelial dysfunction [3,4,16,18,39].

Because environmental and occupational Cd^2+^ exposure varies considerably [40,41,42], two exposure concentrations (40 and 80 ppm) representing commonly used experimental doses were selected [32,43,44,45]. Although impaired ACh-mediated relaxation has been previously reported, the present findings suggest that endothelial dysfunction precedes adrenergic hyperreactivity, as vascular relaxation was impaired at 40 ppm without changes in adrenergic contraction, whereas both responses were altered at 80 ppm.

Phenylephrine-induced contraction remained unchanged in the 40 ppm group but was significantly enhanced following exposure to 80 ppm Cd^2+^, indicating that α-adrenergic receptors only contribute to vascular dysfunction at higher exposure levels [18,46,47]. This hypercontractility was prevented by apocynin, supporting the involvement of NADPH oxidase-derived ROS [17]. Although the molecular basis remains unclear, increased phosphorylation of myosin light chain and activation of p44 MAPK have previously been associated with enhanced Phe-induced contraction [46].

Similarly, U46619 produced greater hypercontractility in HICAD animals, particularly after exposure to 80 ppm Cd^2+^, extending previous observations of TXA_2_-mediated vascular dysfunction in hypertension [19,21]. Importantly, apocynin abolished this response, suggesting that NADPH oxidase-derived ROS also modulate TXA_2_-mediated vascular contraction. Clinical evidence further supports TXA_2_ as a therapeutic target in vascular disease [20,48], reinforcing its potential relevance during chronic Cd^2+^ intoxication.

Oxidative stress was confirmed by the dose-dependent increase in lipid peroxidation markers (MDA and 4-HDA), consistent with previous reports of enhanced oxidative damage following Cd^2+^ exposure [49,50]. ROS overproduction may result from alterations in MAPK signaling, mitochondrial dysfunction [51], or, predominantly, increased NADPH oxidase activity [3,17,27,39,43]. These findings further support NADPH oxidase as a central mechanism linking oxidative stress to hypertension, including pathways involving branched-chain amino acid dysregulation [52,53].

GPx activity increased at 40 ppm Cd^2+^ but was markedly reduced at 80 ppm, suggesting an initial compensatory antioxidant response followed by depletion of antioxidant defenses during sustained oxidative stress [49,50,54,55]. Likewise, GR activity progressively declined with increasing Cd^2+^ exposure, reflecting exhaustion of the glutathione antioxidant system [49]. Together with previous evidence showing that antioxidant enzymes reverse Cd^2+^-induced endothelial dysfunction [17,18], these findings indicate that chronic vascular alterations are primarily mediated by ROS.

Among lipid peroxidation products, 4-hydroxyalkenals (4-HDAs), particularly 4-hydroxynonenal (4-HNE), represent important mediators linking oxidative stress to vascular dysfunction [56]. Increased 4-HDA accumulation reduces NO bioavailability, impairs eNOS activity, enhances vascular smooth muscle contractility, activates MAPK and NF-κB signaling, and further stimulates NADPH oxidase, thereby amplifying oxidative stress [57,58]. These mechanisms position 4-HDAs as a key molecular link between chronic Cd^2+^ exposure, endothelial dysfunction, increased vascular tone, and hypertension.

Moreover, we demonstrated the role of oxidative stress in the progression to hypertension. Although APO is widely used as a pharmacological tool to evaluate the involvement of NADPH oxidase-derived reactive oxygen species, its specificity remains controversial. Previous studies have demonstrated that APO may exert direct antioxidant and free radical-scavenging effects independent of NADPH oxidase inhibition. Therefore, the protective vascular effects observed in the present study following APO incubation may not be exclusively attributable to inhibition of NADPH oxidase activity, but could also result from its general antioxidant properties and its ability to reduce oxidative stress through alternative mechanisms [59,60]. Consequently, the interpretation of the involvement of NADPH oxidase in the observed vascular responses should be approached with caution, and future studies employing more selective inhibitors or molecular approaches are necessary to confirm the specific contribution of NADPH oxidase in Cd^2+^-induced vascular dysfunction.

Although the present study focused primarily on oxidative stress and NADPH oxidase activity in Cd^2+^-induced vascular dysfunction, increasing evidence suggests that the mammalian target of rapamycin (mTOR) signaling pathway may also play a critical role in the pathogenesis of hypertension and vascular injury associated with chronic Cd^2+^ exposure. mTOR is a central regulator of cellular metabolism, growth, autophagy, and inflammatory signaling, and its dysregulation has been strongly associated with endothelial dysfunction, vascular remodeling, and increased vascular tone. Importantly, mTOR signaling has been shown to interact closely with redox-sensitive pathways, particularly through modulation of NADPH oxidase activity and ROS generation. Excessive ROS production may activate mTOR complexes, which in turn amplify oxidative stress and inflammatory responses, creating a self-sustaining cycle of vascular damage. In addition, mTOR activation has been associated with reduced nitric oxide bioavailability, impaired endothelial function, and enhanced vascular smooth muscle cell proliferation, all of which contribute to hypertension development. Emerging evidence also suggests a potential interaction between mTOR signaling and TXA_2_-mediated pathways, since TXA_2_ receptor activation can stimulate intracellular signaling cascades involved in vascular contraction, inflammation, and cellular growth, processes partially regulated by mTOR-dependent mechanisms. Furthermore, mTOR dysregulation may impair autophagic responses required for the removal of oxidatively damaged cellular components, thereby exacerbating Cd^2+^-induced vascular injury. Although mTOR signaling was not directly evaluated in the present study, its established relationship with oxidative stress, NADPH oxidase activation, and vascular dysfunction suggests that this pathway may represent an important mechanistic link and a potential therapeutic target in Cd^2+^-induced hypertension [61,62,63].

Several limitations of the present study should be acknowledged. First, the evaluation of oxidative stress was limited to a restricted panel of biomarkers, including MDA, 4-HDA, GPx and GR. Although these markers provide important information regarding lipid peroxidation and antioxidant defense status, additional biomarkers related to oxidative damage and inflammatory signaling could further strengthen the characterization of the redox imbalance induced by chronic Cd^2+^ exposure.

Additionally, no direct measurements of reactive oxygen species (ROS) production or nitric oxide (NO) bioavailability were performed. Therefore, the involvement of oxidative stress and endothelial NO dysfunction was inferred indirectly from vascular reactivity and oxidative damage markers. Future studies incorporating direct assessment of ROS generation, NO levels, and eNOS activity would provide a more comprehensive understanding of the molecular mechanisms underlying Cd^2+^-induced vascular dysfunction and hypertension.

Although the present study demonstrates enhanced vascular responsiveness to the TXA_2_ receptor agonist U46619 following chronic Cd^2+^ exposure, the molecular mechanisms underlying this effect remain to be elucidated. Specifically, TXA_2_ receptor expression, endogenous TXA_2_ production, and downstream intracellular signaling pathways were not evaluated. Therefore, the observed hypercontractile responses should be interpreted as evidence of increased functional responsiveness rather than definitive proof of enhanced TXA_2_ signaling or receptor upregulation. Likewise, although these findings suggest that TXA_2_ contributes to the vascular dysfunction associated with chronic Cd^2+^ exposure, they do not establish a direct causal role for TXA_2_ in the development of hypertension. Future studies integrating molecular, biochemical, and pharmacological approaches will be necessary to determine whether alterations in TXA_2_ synthesis, receptor expression, or downstream signaling mediate the vascular abnormalities observed in this experimental model.

Overall, the findings regarding the effects of Cd^2+^ on redox modulation indicate that oxidative stress is a central mechanism underlying alterations in vascular contractile and relaxation responses. This effect is closely linked to reduced nitric oxide bioavailability, protein oxidation, altered gene regulation, and the activation of multiple downstream molecular pathways.

Collectively, these findings provide important evidence regarding the vascular mechanisms associated with chronic Cd^2+^ exposure, particularly the involvement of TXA_2_-mediated vascular responses, and may contribute to the understanding and potential treatment of vascular disorders associated with chronic Cd^2+^ intoxication.

## 5. Conclusions

The present study’s results suggest that hypertension induced by chronic Cd^2+^ exposure is closely associated with increased oxidative stress and consequent impairment of endothelial nitric oxide (NO)-mediated vasodilation. The findings further support the hypothesis that chronic Cd^2+^ exposure enhances the activity of NADPH oxidase within the vascular wall, thereby contributing to oxidative imbalance and vascular dysfunction.

In addition, the results indicate that TXA_2_ signalling, as well as α-adrenergic receptor-mediated responses at higher Cd^2+^ concentrations, may participate in the development of Cd^2+^-induced hypertension. Collectively, these findings contribute to a better understanding of the mechanisms underlying hypertension associated with chronic Cd^2+^ exposure and may provide new perspectives for the prevention and treatment of vascular disorders induced by environmental pollutants such as heavy metals.

## Figures and Tables

**Table 1:** Effect of chronic cadmium administration on body weight, blood glucose and systemic blood pressure.

	Body Weight (g)		Glucose (mg/dL)		SBP (mm Hg)	
Week	0	8	0	8	0	8
CTRL	235.3 ± 3.7	419.5 ± 3.1 *	95.7 ± 1.2	98.5 ± 0.9	102.9 ± 1.8	102.4 ± 1.7
Cd^2+^ (40 ppm)	222.6 ± 2.6	380.9 ± 5.8 *	96.1 ± 1.0	110.0 ± 1.0 *	101.5 ± 1.6	121.7 ± 3.2 *
Cd^2+^ (80 ppm)	227.3 ± 4.8	379.3 ± 9.6 *	97.8 ± 0.8	115.5 ± 0.8 *	101.4 ± 1.2	133.1 ± 1.4 *

CTRL: control; Cd^2+^: cadmium; SBP: systolic blood pressure. Results are expressed as mean ± SEM (n = 12). * indicates *p* < 0.05 vs. week 0 (in each parameter). The *p* value was calculated using Student’s *t* test.

**Table 2:** Effect of cadmium on the affinity and potency of vasoactive substances in rat aortas.

ACh						
Condition	CTRL	CTRL/APO	Cd^2+^ (40 ppm)	Cd^2+^ (40 ppm) /APO	Cd^2+^ (80 ppm)	Cd^2+^ (80 ppm) /APO
Emax	0.8 ± 0.03	0.8 ± 0.03	0.5 ± 0.01 *^#+^	0.7 ± 0.07	0.5 ± 0.02 *^#§^	0.7 ± 0.02
pD_2_	6.7 ± 0.10 ^#^	7.2 ± 0.06	6.5 ± 0.12 ^#^	6.7 ± 0.16 ^#^	6.7 ± 0.12 ^#^	6.7 ± 0.14 ^#^
Phe						
Condition	CTRL	CTRL/APO	Cd^2+^ (40 ppm)	Cd^2+^ (40 ppm) /APO	Cd^2+^ (80 ppm)	Cd^2+^ (80 ppm) /APO
Emax	1.0 ± 0.02	0.9 ± 0.23	1.0 ± 0.04	1.0 ± 0.06	1.2 ± 0.02	1.1 ± 0.01
pD_2_	7.0 ± 0.07	6.5 ± 0.10	6.8 ± 0.07	7.0 ± 0.10	7.1 ± 0.05 ^#^	6.9 ± 0.07
U46619						
Condition	CTRL	CTRL/APO	Cd^2+^ (40 ppm)	Cd^2+^ (40 ppm) /APO	Cd^2+^ (80 ppm)	Cd^2+^ (80 ppm) /APO
Emax	1.0 ± 0.03	1.0 ± 0.07	1.4 ± 0.03 *^#+^	1.1 ± 0.08	1.5 ± 0.07 *^#^	1.1 ± 0.07 ^#^
pD_2_	5.4 ± 0.12	5.5 ± 0.07	5.7 ± 0.03	5.4 ± 0.08	6.2 ± 0.09	5.5 ± 0.30

ACh: acetylcholine; APO: apocynin; Emax: maximal effect; pD_2_: log DE_50_; Phe: phenylephrine; U46619: thromboxane A2 analog. Results are expressed as mean ± SEM (n = 6). * indicates *p* < 0.05 vs. CTRL; ^#^ indicates *p* < 0.05 vs. CTRL/APO; ^+^ indicates *p* < 0.05 vs. Cd^2+^ (40 ppm)/APO; ^§^ indicates *p* < 0.05 vs. Cd^2+^ (80 ppm)/APO. The *p* value was calculated using one-way ANOVA coupled to the Bonferroni test.

## Data Availability

The authors confirm that the data supporting the findings of this study are available within the article.
